# Identification of canine papillomavirus by PCR in Greyhound dogs

**DOI:** 10.7717/peerj.2744

**Published:** 2016-12-08

**Authors:** Eman A. Anis, Linda A. Frank, Raquel Francisco, Stephen A. Kania

**Affiliations:** 1 Department of Biomedical and Diagnostic Sciences, University of Tennessee, Knoxville, TN, United States of America; 2 Department of Virology, University of Sadat, Sadat City, Egypt; 3 Department of Small Animal Clinical Sciences, University of Tennessee, Knoxville, TN, United States of America

**Keywords:** Canine papillomavirus, PCR, Greyhound dogs, Corns

## Abstract

**Background:**

Corns are hard protuberances that occur on the digital footpads of Greyhound dogs. The cause of these lesions is unknown and there is little information about them in the veterinary literature. We received anecdotal examples of dog to dog spread of corns suggesting an infectious cause. The aim of this study was to determine if papillomavirus (PV) is associated with Greyhound corns.

**Methods:**

We examined four corns from two unrelated adult Greyhound dogs that resided in Florida and Washington, respectively, for PV by PCR. The samples were obtained by owner coring of two lesions from one dog and laser removal of two lesions from the other dog. Total nucleic acid was extracted and DNA was amplified using two PCR primer sets that have been shown to amplify a broad range of PVs from humans and animals: FAP59/ FAP64 and MY11/ MY09. The DNA sequences were compared with all sequences in GenBank. Formalin-fixed, paraffin-embedded tissue from the footpads of four dogs with other inflammatory dermatoses were also examined.

**Results:**

PV DNA was amplified from all four corn lesions, while no PV DNA was amplified from other tissues. Comparison of the 444-bp sequences amplified by the MY11/ MY09 primers identified two different PVs. One showed 96% nucleotide sequence similarity with the L1 gene of canine PV type 12. The other showed 78% similarity to canine PV type 16 and, therefore, represents a novel PV. In one of the corns, infection by two of the identified PVs was found.

**Discussion:**

These results suggest PV infection could be involved in the pathogenesis of corns in Greyhound dogs.

## Introduction

Footpad lesions, referred to as corns or paw pad keratomas, are hard protuberances that occur on the digital footpads and seem to primarily affect Greyhound dogs. These lesions can be painful and may be associated with lameness and poor performance (Gross et al., 2005). They are mostly seen in middle-aged to older racing or retired racing Greyhound dogs (Balara et al., 2009; Guilliard, Segboer & Shearer, 2010). The majority of corns occur in the center of the more weight bearing digital pads of the front and/or hind feet but can also be found on the metacarpal or metatarsal pads. Diagnosis of corns is usually based on the clinical appearance of circumscribed hyperkeratosis on the paw pad (Gross et al., 2005). The cause of these lesions is unknown and there is very little information about them in the veterinary literature. Theories as to their cause include chronic trauma or pressure, deficiencies in the fatty layer of the pad, scar tissue, foreign bodies or papillomavirus (PV) infection (Guilliard, Segboer & Shearer, 2010).

Papillomaviruses are a group of small, nonenveloped, double-stranded DNA viruses that are epitheliotropic. These epitheliotropic viruses infect a wide range of birds and mammals, including humans, and cause benign cutaneous and mucosal epithelial proliferations called papillomas (warts) (Lancaster & Olson, 1982). The goal of this study was to determine if PV was associated with corns from two Greyhound dogs.

**Figure 1:**
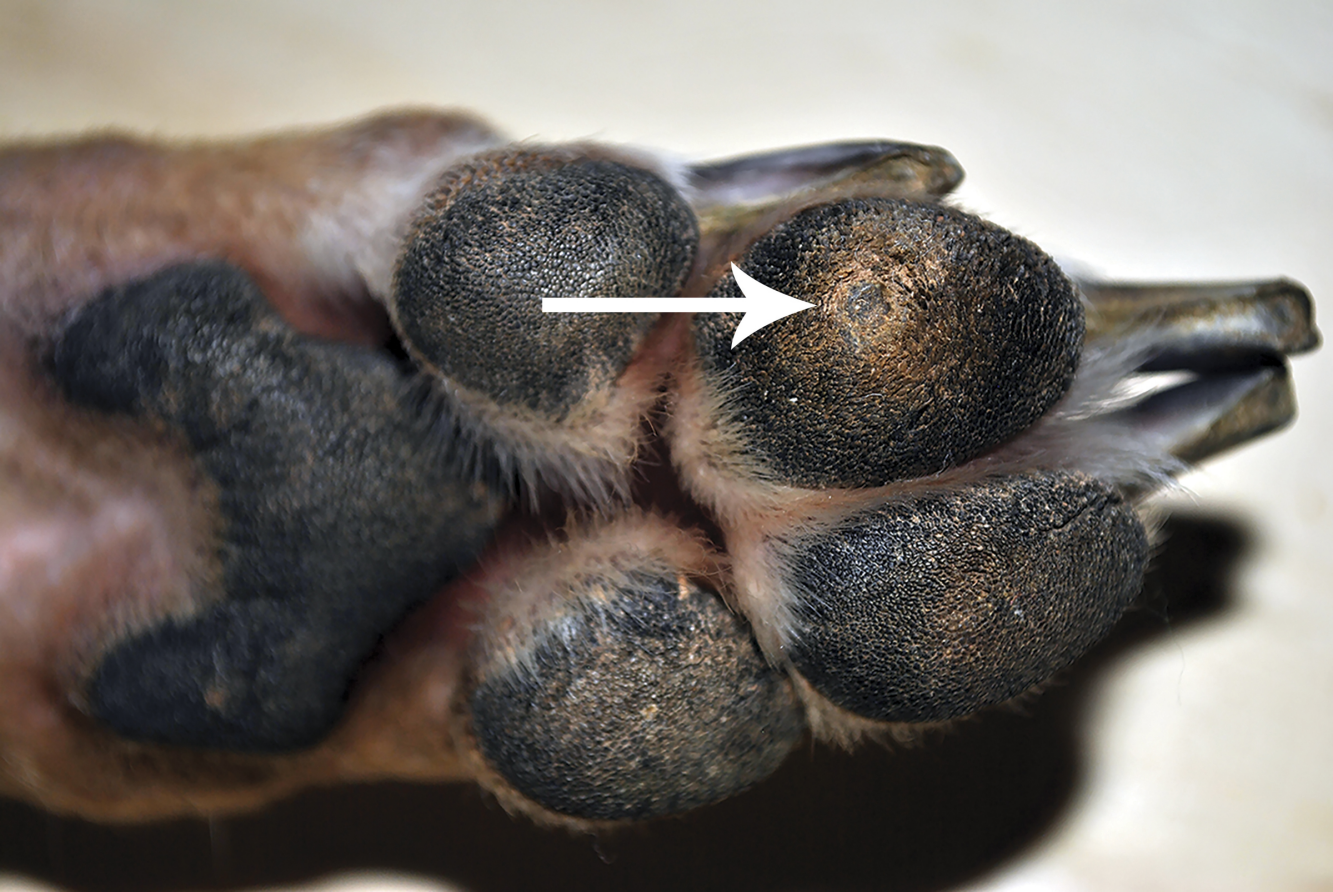
Corn (arrow) on the left front digital pad of digit 3 from Dog 1.

## Materials and Methods

### Samples

Corns were acquired from two Greyhound dogs. Dog 1 is an 8 year old female spayed retired racing Greyhound dog from Florida had a 2–3 year history of corns on digit 3 of both front paw pads (Fig. 1) She had no prior history of corns until 3 months following adoption into a home with another Greyhound dog with corns. Nail trimming equipment that was also used to de-bulk the corns was shared between dogs. One corn from the left and right front feet were provided by the owner for PCR analysis following a routine coring procedure. The samples were processed upon receipt. Dog 2 is a 6.5 year old male castrated retired racing Greyhound dog from Florida who has resided for the past two years with his adopted family in Washington as an only pet, had a one month history of lameness prior to referral. Corns were present on digit 3 of both hind paw pads. There was also a single corn on the central portion of the metatarsal pad of the left hind limb. Two corns from the digital pads were surgically removed by CO_2_ laser. The corns were initially placed in formalin, then transferred to saline and mailed to us for PCR analysis. The samples were processed upon receipt.

### PCR and DNA sequence analysis

Total nucleic acid was extracted from the corn lesions and formalin-fixed, paraffin-embedded (FFPE) tissue scrolls using a commercial kit (DNeasy blood and tissue kit; Qiagen, Valencia, CA, USA) according to the manufacturer’s protocol. The DNA was amplified using two PCR primer sets, FAP59/ FAP64 (Forslund et al., 1999) and MY11/ MY09 (Lurchachaiwong et al., 2009), that have been shown to amplify diverse papillomavirus types from various mammalian tissues. Positive controls for the FAP59/64 primers were DNA extracted from a feline Bowenoid in situ carcinoma, while no template DNA (water only) was added to the negative controls. The MY11/MY09 primer set did not amplify a feline papillomavirus control DNA template. PCR mixtures contained 1.5 µL each of forward and reverse primers (concentration: 5 µM), 6.5 µL of nuclease-free water, 12.5 µL of Taq premix (rTaq^®^; Takara Bio, Otsu, Shiga, Japan), and 5 µL of DNA template. The same reaction conditions previously described for the FAP59/64 primers (Forslund et al., 1999) were used for all primer sets. The PCR products were analyzed by electrophoresis in a 1.4% agarose gel containing ethidium bromide. PCR products from three lesions were cloned using the TOPO TA Cloning kit (Invitrogen, Carlsbad, CA, USA). Five clones from each PV-positive sample were isolated and sequenced. To sequence PCR products, primers were digested using ExoSAP-IT (USB, Cleveland, OH, USA), according to the manufacturer’s instructions. Samples were sequenced at the University of Tennessee Molecular Biology Resource Facility using Sanger sequencing with an ABI prism dye terminator cycle sequencing reaction kit (Perkin Elmer Inc, Foster City, CA, USA) and a capillary electrophoresis instrument (ABI 373 DNA, Perkin Elmer Inc, Foster City, CA, USA). The PCR product sequences were compared to sequences from GenBank using the basic local alignment search tool (BLAST; http://www.ncbi.nlm.nih.gov/blast/Blast.cgi) and in multiple sequence alignments using the Clustal W alignment algorithm with the slow-accurate option (DNASTAR MegAlign version 13.0.0).

To confirm the etiologic link of PV with corns, FFPE tissue from the footpads of four other dogs with various inflammatory diseases including pemphigus foliaceus, hepatocutaneous syndrome, split paw pad disease, and parakeratosis with bacterial colonization were also examined. The quality of the extracted nucleic acid of all the control samples was confirmed using a 4,200 TapeStation instrument. The presence of sufficient DNA for amplification was determined by routine canine GAPDH PCR. The GAPDH amplification reaction was performed as follows: 95 °C for 2 min, 45 cycles of 95 °C for 10 s, 60 °C for 40 s, 72 °C for 30 s. Furthermore, our laboratory was able to successfully amplify PV DNA from FFPE samples in previous studies (Anis et al., 2010; Newkirk et al., 2014).

## Results

**Figure 2:**
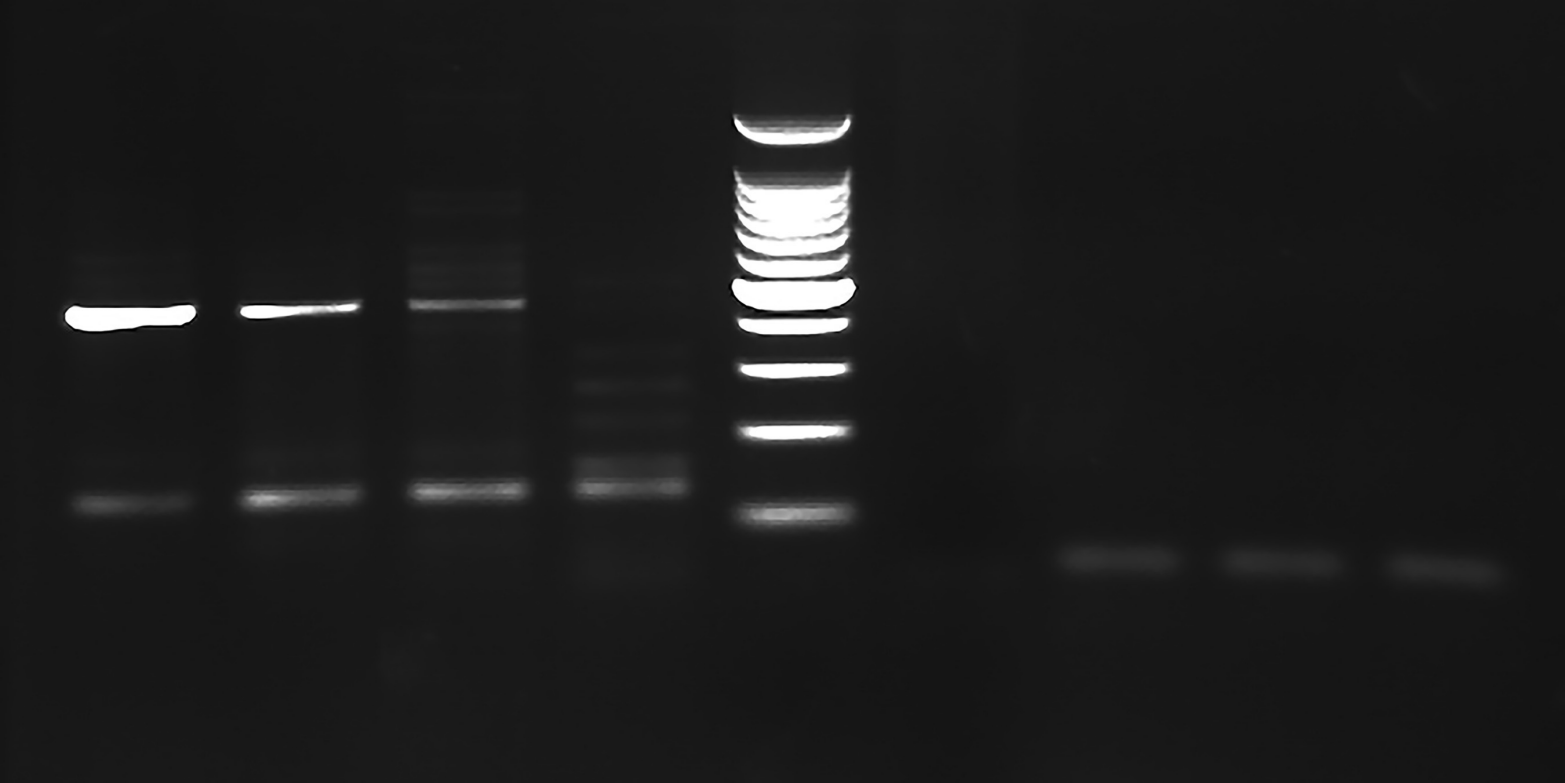
PCR gel. PCR amplification from Greyhound dog corns using MY11/MY09 primer set. Lanes 1–3 corn lesions from dog 1, lane 4 corn lesions from dog 2, lane 5 molecular mass marker (100 bp Plus DNA Ladder, Fisher Scientific), lane 6 negative control with no DNA added and lanes 7–9 negative samples. The size of the PCR products are estimated from the gel to be approximately 450 bp.

**Figure 3:**
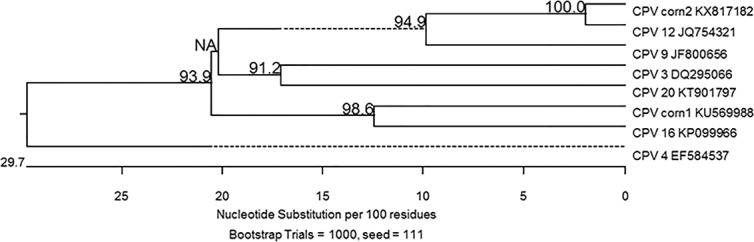
Algorithm with bootstrapping. The partial capsid L1 gene sequences obtained in this study from corn 1 (CPV corn 1 KU569988) and corn 2 (CPV corn 2 KX817182) were compared to the closest sequences available in GenBank using the Clustal W alignment algorithm with bootstrapping. Each sequence is identified with its GenBank accession number. Dashed lines indicate a negative branch length that results from indels.

Both primer sets were able to amplify papillomavirus DNA from all four corn lesions, while no PV DNA was amplified from the other examined tissues (Fig. 2). Multiple products of different sizes were produced from corn 2 necessitating the removal of the approximately 450 bp product from the gel for analysis and cloning. Comparison of the 444 bp sequences amplified by the MY11/ MY09 primers identified two different PVs. One PV amplified from both dogs had 96% nucleotide sequence similarity with the L1 gene nucleotide sequence of the recently reported canine papillomavirus (CPV) type 12 (GenBank accession No. JQ754321) and has been depositied in Genbank with accession number KX817182. The other viral DNA was amplified and cloned only from Dog 1. It revealed the greatest similarity to CPV type 16 (GenBank accession No. KP099966) with 78% similarity. Both sequences aligned most closely with other canine papillomaviruses (Fig. 3). Although only a segment of the entire L1 gene was sequenced, this sequenced segment suggests a putative novel PV (GenBank accession No. KU569988). In one of the examined corn lesions from Dog 1 there was a double infection by the two identified CPV.

## Discussion

These results are the first evidence that Greyhound dog corns may be associated with PV. Attempts to link the condition to PV in the past have been unsuccessful. Histologically, the lesions are characterized by well-defined conical hyperkeratosis that project above the skin surface with no evidence of viral cytopathology or inflammation (Gross et al., 2005). A previous study in which immunohistochemistry and PCR were performed on paraffin embedded tissue obtained from six Greyhound dogs with corns failed to identify any PV DNA (Balara et al., 2009). Although that study used the same primer set used in the current study, the type of samples as well as the annealing temperature were different. The sensitivity and specificity of the PCR may be affected by many factors such as type of sample, DNA extraction procedure, purity of the sample DNA and PCR setting. In the current study an annealing temperature of 50 °C was used instead of 55 °C. This lower annealing temperature can amplify a broader range of DNA templates (Ishii & Fukui, 2001). Furthermore, in the previous study the immunohistochemistry was done using a single monoclonal antibody directed against human papillomavirus L1 capsid epitope (Balara et al., 2009). Although this antibody was able to detect various PV including HPV-1, 6, 11, 16, 18, and 31, its reactivity with all types has not been determined. In addition, L1 capsid proteins are not expressed in all papilloma associated lesions, explaining possible false negative results (Yemelyanova et al., 2013).

Papillomaviruses are an established cause of skin disease in dogs. They are circular, double-stranded DNA viruses with a genome of approximately 8 kb pairs. Papillomaviruses are classified into genus, species, and type based on the nucleotide sequence of the L1 open reading frame. The L1 gene is highly conserved, and a new putative PV type is considered when the L1 nucleotide sequence is at least 10% different from other PV types (De Villiers et al., 2004). Currently 16 types of CPVs have been identified (Luff et al., 2015). Canine PV type 12, which was isolated from three of the corns in the present study, has been isolated and sequenced from a solitary pigmented plaque on a mixed breed bloodhound (Zhou et al., 2015).

In order to support the etiologic link of PV with corns in the present study, FFPE tissue from the footpads of four other dogs with various inflammatory diseases were also examined. No papillomavirus DNA was amplified from these examined lesions. Detecting PV within a lesion, however, does not prove a causal relationship. Further study is needed to strengthen the etiological link of PV with corns by performing IHC and/or *in situ* hybridization to localize PV protein or DNA in a section of the corn lesions. Also, more corn lesions need to be examined for the presence of PV.

## Conclusions

These results suggest that PV infection could be related to the pathogenesis of corns in Greyhound dogs. Understanding the cause of this disease may lead to a more successful treatment outcome.

## Additional Information and Declarations

### Data Availability

The following information was supplied regarding data availability: GenBank: KU569988, KX817182
